# Levofloxacin-Induced Fuchs Syndrome: A Rare Atypical Stevens–Johnson Syndrome Variant With a Comprehensive Literature Review

**DOI:** 10.1155/carm/9944674

**Published:** 2025-11-20

**Authors:** Aseel Abuhammad, Mohammed Ayyad, Maram Albandak, Motaz Natsheh, Saed I. Y. Attawna

**Affiliations:** ^1^Faculty of Medicine, Al-Quds University, Jerusalem, State of Palestine; ^2^Department of Medicine, Rutgers New Jersey Medical School, Newark, New Jersey, USA; ^3^Department of Medicine, University of Toledo, Toledo, Ohio, USA; ^4^Department of Medicine, Al-Ahli Hospital, Hebron, State of Palestine

**Keywords:** case report, Fuchs syndrome, genital ulcer, levofloxacin, mucosal ulcerations, mucositis, Stevens–Johnson syndrome

## Abstract

**Background:**

Fuchs syndrome, also known as atypical Stevens–Johnson syndrome (SJS), is a rare variant of SJS that primarily affects the mucosae of the mouth, eyes, and genitalia with minimal to no skin involvement. This case is notable due to the absence of skin manifestations and the critical need for accurate diagnosis and appropriate management, especially following drug administration.

**Case Presentation:**

We report a case of a 22-year-old male patient who presented with severe oral ulcers, causing difficulty in talking and swallowing. The patient was recently treated for an upper respiratory tract infection with levofloxacin. Shortly thereafter, he developed oral and genital ulcers, as well as conjunctivitis. Upon admission, various differential diagnoses including autoimmune and infectious were considered but subsequently ruled out. As symptoms worsened, a biopsy of the affected mucosa was performed and confirmed the diagnosis of SJS. The patient was subsequently treated with corticosteroids resulting in rapid improvement of his symptoms.

**Conclusions:**

This case highlights the importance of considering Fuchs syndrome as a potential cause of mucositis following drug administration, particularly in the absence of cutaneous lesions. Accurate diagnosis and prompt treatment are crucial for patient recovery.

## 1. Introduction

Stevens–Johnson syndrome (SJS) is a severe, potentially life-threatening disorder that predominantly affects the mucous membranes and skin [1]. Cutaneous involvement is a hallmark of SJS, developing in more than 90% of cases [1]. Patients typically present with a prodrome of fever and flu-like symptoms, followed by the rapid onset of erythematous macules, which can progress to blistering and mucosal involvement. SJS can lead to significant morbidity and mortality, necessitating prompt diagnosis and treatment.

Fuchs syndrome, also known as atypical SJS, represents a rare variation of SJS. It is characterized by the involvement of mucosal membranes without significant skin manifestations [2]. This variant primarily affects the mouth, eyes, and genitalia. Fuchs syndrome is less frequently encountered in clinical practice compared to classic SJS and is most commonly reported in children with *Mycoplasma pneumoniae* infection [3]. The pathophysiology of Fuchs syndrome, like that of SJS, involves immune-mediated damage, often triggered by infections or medications.

Despite the known association with *Mycoplasma pneumoniae*, Fuchs syndrome can also occur following exposure to certain medications. In this context, the condition presents a diagnostic challenge due to the absence of typical cutaneous signs, necessitating a high index of suspicion and thorough clinical evaluation. The rarity of this presentation highlights the importance of recognizing and differentiating Fuchs syndrome from other causes of mucositis [4].

In this study, we present a rare case of mucosal-predominant SJS (Fuchs syndrome) in a young male patient who developed mucosal ulcerations of the mouth, esophagus, genitalia, and conjunctiva, without any accompanying skin lesions. This case followed exposure to the antibiotic levofloxacin and exhibited no association with *Mycoplasma pneumoniae* infection. Additionally, we also review the existing literature on Fuchs syndrome following drug exposure, highlighting the importance of considering this diagnosis in similar clinical scenarios.

## 2. Case Description

A 22-year-old male presented to the emergency department with fever, chills, bilateral eye redness, and decreased oral intake for four days. He had initially been evaluated at an outpatient clinic, where he was prescribed acyclovir and antifungal mouthwash, but he was unable to take them due to severe odynophagia. His symptoms began 10 days prior with sore throat, rhinorrhea, and fatigue, which progressively worsened. Over the past few days, he developed new-onset chest pain, shortness of breath, productive cough, and persistent chills.

On presentation, his vital signs were notable for a respiratory rate of 14 breaths per minute, heart rate of 118 beats per minute, and oxygen saturation of 96% on room air. A chest X-ray revealed multifocal pneumonia, prompting initiation of levofloxacin and dexamethasone. However, within days of starting levofloxacin, he developed extensive oral ulcerations, worsening dysphagia, and persistent chest pain. Upon further questioning, he also reported a history of painful genital lesions, though no associated skin rash was present. Initial laboratory studies on admission are summarized in Table 1.

**Figure 1:**
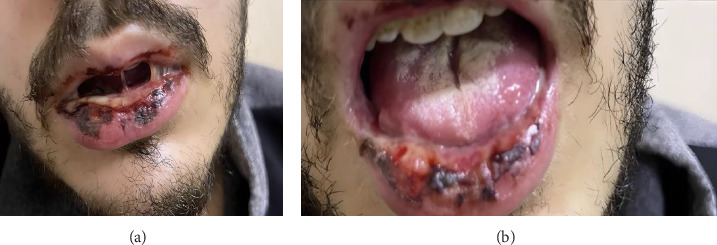
Diffuse oral ulcerations observed in a 22-year-old patient diagnosed with Fuchs syndrome (a, b).

**Figure 2:**
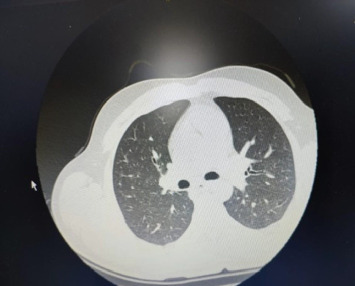
Chest CT scan revealing no signs of infection or notable abnormalities, suggesting the absence of pulmonary involvement.

**Figure 3:**
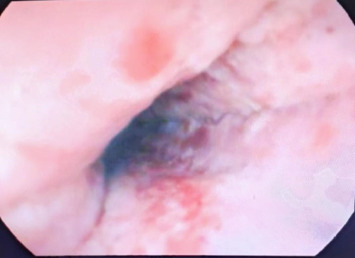
Inflammation and ulcerations localized to the mucosa of the esophagus, indicative of Fuchs syndrome.

On admission, a physical examination revealed severe oral ulcerations involving the hard and soft palate and tonsils (Figure 1). Additionally, the patient had injected conjunctiva and multiple genital lesions. A comprehensive infectious workup, including testing for *Cytomegalovirus*, Epstein–Barr virus, HIV, COVID-19, and a full respiratory panel, was negative. A full septic workup was also negative. Laboratory findings revealed leukocytosis with a left shift and elevated inflammatory markers. A CT scan of the chest showed evidence of resolving pneumonia (Figure 2). Given concerns for a fungal infection or superimposed bacterial infection, the patient was started on intravenous fluconazole and broad-spectrum antibiotics. Despite this treatment, his symptoms continued to worsen, with progressive oral ulceration and dysphagia. He also experienced persistent chest pain, raising concerns for an alternative diagnosis. An upper endoscopy was performed, revealing diffuse whitish plaques with surrounding erythema in the oropharynx and esophagus, as well as a small hiatal hernia (Figure 3). Despite continued antimicrobial therapy, there was no clinical improvement, prompting further evaluation.

Given the diagnostic complexity and the absence of skin involvement, the differential diagnosis included erythema multiforme major, fixed drug eruption, Behçet's disease, and primary herpetic infection. Erythema multiforme major was ruled out due to the lack of targetoid lesions and skin findings. Fixed drug eruption was considered less likely due to the multifocal mucosal involvement and lack of recurrence with repeated exposure. Serologic and microbiologic testing for herpes simplex virus, Candida, and other infections was negative.

A dermatology consultation was obtained, given the patient's worsening mucosal involvement and concern for atypical SJS. The dermatologist recommended a mucosal biopsy from the lower lip to confirm the diagnosis and rule out other potential causes, including autoimmune disease, drug-induced eruptions, and infections. Based on clinical suspicion, the patient was initiated on high-dose systemic corticosteroids (methylprednisolone 250 mg IV for three doses) and topical steroid therapy. While a drug-induced lymphocyte stimulation test (DLST) was not performed due to resource limitations, the temporal relationship between levofloxacin administration and symptom onset, along with exclusion of infectious and autoimmune causes, strongly supported a drug-induced etiology.

**Figure 4:**
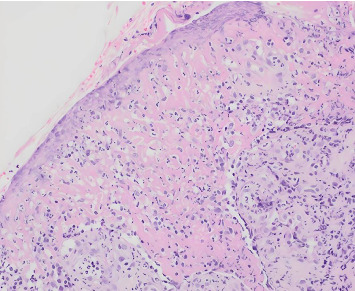
Histological examination (20 × magnification) displaying full-thickness epithelial necrosis, ulceration, and detachment. Stained with hematoxylin and eosin, with an original magnification of 20 ×.

Following steroid treatment, the patient showed marked improvement in his oral and genital lesions. He was discharged in stable condition with plans for outpatient follow-up. One week post-discharge, biopsy results confirmed full-thickness epidermal necrosis, ulceration, and detachment, consistent with SJS (Figure 4). Special staining with periodic acid-Schiff and Grocott–Gomori methenamine silver was negative for fungal elements. At follow-up, the patient had fully recovered, with no residual mucosal ulcerations and normalization of inflammatory markers.

## 3. Discussion

### 3.1. SJS

SJS is a serious adverse reaction to drugs characterized by inflammatory changes in both mucosa and skin. SJS was initially reported in 1922 (by Albert Mason Stevens and Frank Chambliss Johnson) in two pediatric patients [1]. SJS involves less than 10% of the body surface area. In addition, toxic epidermal necrolysis (TEN) causes widespread mucocutaneous lesions that cover more than 30% of the surface area (Table 2) [4]. It is essential to consider that the body surface area affected by detached skin serves as a crucial clinical differentiator among SJS, overlap SJS/TEN, TEN, and atypical SJS [5].

The clinical manifestation of SJS usually affects the mucosa, which presents with painful erosions that may include the mouth, conjunctiva, nasal cavity, urethra, vagina, gastrointestinal tract (GIT), respiratory tract, or any combination of them. Moreover, 90% of cases had mucous involvement [4]. Affected skin in SJS is commonly a diffuse erythematous macule; in addition, it is found to be purpuric with necrotic centers and overlying blistering. Ocular complications have been common in SJS patients in the long term. However, anterior uveitis, iritis, keratitis, and conjunctivitis have been less common [4]. The GIT, especially the esophagus, was reported in SJS, and esophageal webbing or stricture can develop in these patients [6, 7]. Severe esophageal involvement has been less common. In these cases, upper endoscopy revealed a whitish plaque with erythema or inflamed mucosa [6]. A retrospective study of GIT involvement in SJS showed that 90% of patients among the 20 endoscopic procedures performed (18 of the 20 cases) had abnormal findings, with ulceration being the most common (50% of patients). Nine cases had an ulceration in the esophagus, 4 cases had gastroduodenal ulceration, and 1 case had a colon ulceration. Mucosal inflammation was reported in 45% of patients, and esophageal stricture was reported in 15% of cases [6]. In a systematic review including 381 cases, clinical characteristics during the acute phase of SJS showed mucous membrane involvement in 87.4% of patients and visceral organ involvement in 14.7% of cases [8].

### 3.2. Fuchs Syndrome

Fuchs syndrome refers to SJS limited to mucosal involvement without significant skin lesions. It mostly affects oral, ocular, and genital mucosae. Fuchs syndrome has different terms in the literature, including atypical SJS, incomplete SJS, or *Mycoplasma pneumoniae*–associated mucositis [9]. Differentiating Fuchs syndrome from other mucosal-predominant conditions can be challenging. In our case, erythema multiforme major was ruled out due to the absence of classic target lesions and skin involvement. Fixed drug eruption was excluded given the absence of recurrence upon reexposure and the multifocal nature of mucosal involvement. Infectious causes were ruled out with comprehensive microbiologic testing. Autoimmune diseases such as Behçet's syndrome were considered, but the clinical course and biopsy findings were not consistent. While a DLST could have further supported the causative role of levofloxacin, this was not performed due to limited availability. Nonetheless, the close temporal relationship between drug exposure and symptom onset, combined with the exclusion of alternative etiologies, reinforced the diagnosis.

Compared to SJS, the majority of cases with atypical SJS have better prognosis and recovery time [10]. To the best of our knowledge, a few cases with Fuchs syndrome were reported following drug exposure without evidence of *Mycoplasma pneumoniae* infection (Table 3) [3, 7, 11–17]. In previous reported cases, the mean age of the respective patients was 32.1 years, and the median age was 26.5 years. All patients had stomatitis or mouth mucosa involvement; 8 patients had ocular involvement; two patients had a genital ulcer; two patients had esophagitis; one case had urethritis; and one case had laryngitis.

As far as we are aware, esophagitis is uncommon in SJS and less common in Fuchs syndrome, particularly in patients who do not have *M. pneumoniae* [18]. Our patient's diagnosis was delayed because the absence of skin lesions made the presentation atypical. Initially, fluconazole was used to treat a suspected fungal infection, but no improvement was seen. Therefore, looking into alternative causes was essential. In such situations, a mucosa biopsy is helpful, and consulting a dermatologist can also aid in the diagnosis. Diffuse mucositis affecting the conjunctiva, oral mucosa, esophagus, and genitalia was evident in our case. This presentation of Fuchs syndrome following levofloxacin was uncommon.

## 4. Conclusions

This case is notable for two distinct reasons. First, it represents an uncommon example of drug-induced Fuchs syndrome in the absence of *Mycoplasma pneumoniae* infection. Second, it demonstrates significant gastrointestinal involvement—including esophageal disease—which remains rarely reported in both classic SJS and its atypical mucosal-predominant variants. The unusual combination of mucosal involvement across the oral cavity, conjunctiva, genitalia, and esophagus, without any accompanying skin lesions, contributed to a delayed diagnosis and highlights the diagnostic challenges posed by this presentation. Given these factors, clinicians should maintain a high index of suspicion for Fuchs syndrome in patients who develop multifocal mucositis after recent drug exposure, even when cutaneous findings are absent. Early recognition, timely dermatologic evaluation, and prompt initiation of immunosuppressive therapy are critical to prevent progression and reduce the risk of long-term complications such as esophageal stricture.

## Figures and Tables

**Table 1:** Laboratory results of the patient on admission.

Laboratory test	Value	Normal values
*Complete blood count*		
WBC count	11.2 × 10^9^/L	(4.5–11.0 × 10^9^/L)
Hemoglobin	13.2 g/dL	12.1–15.1 g/dL
Platelets	430 K/UL	140–400 K/uL
Neutrophils	76.8%	40%–60%
Lymphocytes	15.9%	20%–40%

*Serum chemistry*		
Na	141 mEq/L	135 to 145 mEq/L
K	4.0 mEq/L	3.5 to 5.5 mEq/L
BUN	12 mg/dL	6–24 mg/dL
Creatinine	0.7 mg/dL	0.6–1.1 mg/dL
Total bilirubin	0.7 mg/dL	0.1–1.2 mg/dL
Direct bilirubin	0.3 mg/dL	< 0.3 mg/dL
ALP	68 IU/L	44–147 IU/L
AST	18 IU/L	< 40 IU/L
ALT	15 IU/L	19–25 IU/L

*Immunology workup*		
IgM antibodies	89	22–240 mg/dL
IgG antibodies	1197	540–1822 mg/dL
IgA antibodies	344	63–484 mg/dL
IgE antibodies	38.8	< 100
CRP titer	170 mg/dL	6 mg/L
ESR	95 mm/hour	0–15 mm/hour

*Infectious workup*		
HIV	Negative	Negative
EBV-IgG	75.6	< 0.09 Negative > 0.21 Positive
EBV-IgM	0.15	< 0.5 Negative > 1.0 Positive
COVID-19 antigen	Negative	Negative
Influenza A	Negative	Negative
Influenza B	Negative	Negative
Blood culture	NO growth	NO growth
Sputum culture	NO growth	NO growth
Urine culture	NO growth	NO growth

*Note:* Na: sodium; K: potassium; ALP: alkaline phosphatase; AST: aspartate aminotransferase; ALT: alanine transaminase; COVID-19: coronavirus disease 2019.

Abbreviations: BUN, blood urea nitrogen; CRP, C-reactive protein; EBV, Epstein–Barr virus; ESR, erythrocyte sedimentation rate; HIV, human immunodeficiency virus; WBC, white blood cells.

**Table 2:** Clinical characteristics used to distinguish between SJS, SJS-TEN overlap, TEN, and atypical SJS.

Classification	Clinical feature
SJS	Lesions involve less than 10% of the body's surface area of detached skin
Overlap Stevens–Johnson syndrome/toxic epidermal necrolysis (SJS/TEN)	Lesions involve more than 10% and less than 30% of the surface area
Toxic epidermal necrolysis (TEN)	Lesions that cover more than 30% of the surface area
Atypical SJS	Absence or a few skin lesions present

*Note:* Atypical SJS: atypical Stevens–Johnson syndrome [4].

Abbreviations: SJS, Stevens–Johnson syndrome; SJS/TEN, Stevens–Johnson syndrome/toxic epidermal necrolysis; TEN, toxic epidermal necrolysis.

**Table 3:** Cases of Fuchs syndrome associated with drug exposure.

Study ID	Sex/age (year)	Description	Drug's name	Treatment
Our case	M/22	Stomatitis, conjunctivitis, esophagitis, genital lesions	Levofloxacin	mPSL 250 mg/day × 3 days and topical steroid therapy
Strawn et al. [3]	M/15	Stomatitis, conjunctivitis, urethritis	Duloxetine	Oral acyclovir and cefdinir
Anders et al. [7]	M/14	Stomatitis, episcleritis	Azithromycin	NR
Alsulami et al. [11]	F/28	Stomatitis, conjunctivitis	Azithromycin	Hydrocortisone 100 mg/day
Sumida et al. [12]	F/31	Stomatitis, conjunctivitis	Lamotrigine	Steroid pulse (mPSL 1 g/day x 3 days) followed by intravenous PSL 60 mg/day
Ishiguro et al. [13]	F/58	Stomatitis, vulvar erosions	Tizanidine and meloxicam	PSL 70 mg/day
Canter and Smith [14]	M/25	Stomatitis, conjunctivitis	TMP-SMX	mPSL
Dong et al. [15]	M/38	Stomatitis, conjunctivitis, pharyngitis, laryngitis	Sulfasalazine or diclofenac sodium	Etanercept
Techasatian et al. [16]	M/6	Stomatitis	Carbamazepine	PSL 1 mg/kg/day
Ishikawa et al. [17]	F/84	Stomatitis, conjunctivitis, esophagitis, gastritis	TMP-SMX	mPSL 1 g/day and oral PSL

*Note:* TMP-SMX: trimethoprim/sulfamethoxazole; F: female; M: male; PSL: prednisolone; mPSL: methylprednisolone.

Abbreviation: NR, not reported.

## Data Availability

The datasets used during the current study are available from the corresponding author on reasonable request.
